# Molecular detection and sequencing of beet necrotic yellow vein virus and beet cryptic virus 2 in sugar beet from Kazakhstan

**DOI:** 10.3389/fmicb.2024.1461988

**Published:** 2024-11-12

**Authors:** Alexandr Pozharskiy, Aruzhan Mendybayeva, Ruslan Moisseyev, Marina Khusnitdinova, Gulnaz Nizamdinova, Dilyara Gritsenko

**Affiliations:** ^1^Laboratory of Molecular Biology, Institute of Plant Biology and Biotechnology, Almaty, Kazakhstan; ^2^Department of Molecular Biology and Genetics, Al-Farabi Kazakh National University, Almaty, Kazakhstan

**Keywords:** BNYVV, *Polymyxa betae*, rhizomania, BCV2, LAMP

## Abstract

**Introduction:**

Beet necrotic yellow vein virus (BNYVV) is a common viral pathogen that causes considerable economic loss globally. In the present study, a commercial realtime PCR test system and custom loop mediated amplification primers were used to detect the virus in asymptomatic sugar beet samples.

**Methods:**

A total of 107 of 124 samples tested positive for the presence of the A type BNYVV coat protein gene. Near complete sequences of RNA-3 and RNA-4 were obtained using reverse transcription, followed by nanopore sequencing of 14 samples.

**Results and discussion:**

A comparison with available sequences, including previously published isolates Kas2 and Kas3 from Kazakhstan, identified RNA-3 as similar to such of the P-type isolates Puthiviers and Kas3. RNA-5 was not detected using real-time PCR or cDNA amplification. Unique variable sites were identified in the p25 protein sequence translated from RNA-3. Another virus, beet cryptic virus 2 (BCV2), was identified and sequenced in samples infected with BNYVV. With 85.28% genome coverage, the identified BCV2 samples were very similar to the previously reported isolates from Hungary and Germany

## 1 Introduction

According to data from the Food and Agriculture Organization of the United Nations and national statistics, sugar beet production has declined in Kazakhstan, both in terms of decreasing harvest area and average yield per hectare (Food and Agriculture Organization of the United Nations, 2021; Bureau of National Statistics, 2023). One known problem leading to the decrease in sugar beet production is the spread of pests and diseases (Khusnitdinova et al., 2023). Rhizomania, or “root madness,” caused by beet necrotic yellow vein virus (BNYVV), is one of the most dangerous diseases of sugar beet, which appears mostly in those regions of Eurasia, Africa, and the United States, where sugar beet is grown (EPPO, 2024). Characteristic symptoms of this disease include extensive proliferation of thin rootlets (“bearded root”), abnormal upright above-ground growth, wilting, and leaf chlorosis (Harveson, 2021; Tamada and Baba, 1973). BNYVV reduces production traits, such as root yield and sugar content in sugar beet (Fasahat et al., 2024). In Kazakhstan, rhizomania is widespread in the regions where sugar beet is grown and is responsible for up to 70–80% yield losses, and substantially reduces the sugar content (Maui et al., 2015). Because sugar beets are one of the main sugar sources globally (Rush et al., 2006) and the primary source in Kazakhstan (Abekova et al., 2022), the damage caused by BNYVV has a considerable economic impact.

BNYVV, belonging to species *Benyvirus necrobetae*, included into *Benyviridae* family, *Riboviria* [according to ICTV Taxonomic report (Lefkowitz et al., 2018)], is a multipartite RNA virus consisting of four to five separate rod-shaped nucleoprotein particles of lengths 80–390 nm, corresponding to encapsidated genomic RNA segments (Tamada, 2016). RNA1 and RNA2 are the longest segments with lengths of 6,746 and 4,612 bases, respectively, and are essential for viral replication and infection (Peltier et al., 2008). RNA-1 contains one open reading frame (ORF) encoding the replicase polyprotein p237, consisting of four functional domains: methyltransferase (MTF), helicase (HEL), papain-like protease (PRO), and polymerase (POL) domains. The PRO is responsible for autocatalytic cleavage, which releases a separate polymerase protein (66 kDa) (Hehn et al., 1997). RNA-2 contains six ORFs encoding coat protein (CP), the standard p21, and the extended p75 resulting from an occasional amber stop-codon readthrough (Haeberle et al., 1994), “triple gene block” responsible for cell-to-cell movement (Erhardt et al., 2005), and cysteine-rich protein p14, a silencing suppressor, regulating accumulation and the long-distance transport of the virus (Chiba et al., 2013; Gilmer, 2016). *In vitro* experiments using plant inoculation demonstrated that the RNA-1 and RNA-2 segments are necessary and sufficient for the replication of BNYVV inside host cells (Tamada and Abe, 1989). The CP contains three variable amino acid residues at positions 62, 103, and 172, which are used to classify BNYVV into subtypes A and B (Schirmer et al., 2005). RNA-3, RNA-4, and RNA-5 are smaller (1773, 1,467, and 1,350 bases, respectively) and are important for viral transmission and symptom expression in plants (Peltier et al., 2008). RNA-3 contains a single ORF encoding protein of 25 kDa responsible for the expression of rhizomania symptoms (Tamada et al., 1999) and the resistance breaking ability of the virus (Bornemann et al., 2015). A typical feature of the p25 protein is a variable amino acid tetrad at positions 67–70 under strong selection pressure, and is thus considered to be involved in evasion of the host immune response (Schirmer et al., 2005). RNA-4 encodes a 31 kDa multifunctional protein that plays a role in efficient vector transmission, enhanced symptom expression, and root-specific silencing suppression (Rahim et al., 2007). RNA-5 is an additional segment present exclusively in BNYVV types J (Japan) (Kiguchi et al., 1996) and P (named after Pithivier town, France) (Koenig et al., 1997). This RNA species encodes a single 26 kDa protein with a synergistic effect on disease development and transmission (Peltier et al., 2008; Tamada, 2016). J- and P-type BNYVV can also be characterized by specific tetrad motif 67–70 in p25 protein (Peltier et al., 2008); based on this, P-like isolates lacking RNA-5 have been identified in Kazakhstan (Koenig and Lennefors, 2000) and Iran (Mehrvar et al., 2009).

Transmission of BNYVV occurs via the plasmodiophorid *Polymyxa betae* (Abe and Tamada, 1986), previously classified as a fungus (Braselton, 1995). The pathosystem of the virus and its vector is highly persistent in soil as the virus can retain its activity within the dormant spores (cysts) of *P. betae* over 15 years (EPPO, 2024; Scholten and Lange, 2000). Notably, the sugar beet plant, *P. betae* and BNYVV are in complex interactions at molecular level; while, *P. betae* attenuates the plant’s defense response against the virus, the virus accelerates the life cycle of the plasmodiophorid and promotes formation of the resting spores, thus ensuring its own conservation and propagation (Decroës et al., 2022). The presence of *P. betae* infestation, indicated by clusters of resting spores (sporosori) within plant roots, is a prerequisite for BNYVV infection. However, the protist itself is not always viruliferous (Hugo et al., 1996).

An adequate response to the increasing diversity of pests and diseases requires the development of accurate, quick, and convenient diagnostic and analytical tools, as well as extensive studies to understand the distribution and evolution of pathogens. This is especially important in the case of viral infections, for which no efficient methods other than timely detection and elimination of infected plants are available. A variety of qualitative and quantitative polymerase chain reaction (PCR) assays, as well as sequencing analyses of nucleic acids, have become the gold standard in such studies because of their robustness and potential precision in the identification and characterization of plant pathogens.

Besides disease inducing viruses such as BNYVV, beet soilborne virus, beet mosaic virus, etc., there are known beet viruses belonging to the plant cryptic viruses (genus *Deltapartitivirus* of *Partitiviridae* family, *Riboviria*). This group of viruses is characterized by the symptomless persistence in infected plants and thus remains out of attention of researchers. However, they pose interest because of some of their properties like double-stranded RNA (dsRNA) genome composition and possible interference with analytical assays for other plant viruses (Boccardo et al., 1987). In beet, three serologically and genetically distinct cryptic viruses are known: beet cryptic virus 1 (BCV1), BCV2, and BCV3 (Xie et al., 1994). Previously, we have found some reads classified as BCV2 in exploratory RNA sequencing experiments on sugar beet isolated from the field in Kazakhstan (unpublished data). BCV2 (*Deltaprtitivirus duobetae*, according to ICTV) have genome consisting of three dsRNA segments, with dsRNA-1 encoding RNA-dependent RNA-polymerase, dsRNA-2a and dsRNA-2b encoding unidentified viral proteins (reference genome assembly ASM286851v1 (RefSeq ID GCF_002868515.1)—Szego, Enunlu, and Lukacs, unpublished). To date, BCV2, as well as BCV1 and BCV3, remain unstudied. Notwithstanding, they may potentially affect plant health under certain growing conditions (Xie et al., 1994).

In the present study, a real-time PCR assay was used for the differential detection of three main viral subtypes of BNYVV, A, B, and P, in sugar beet roots collected from two sugar beet fields in the Zhetysu region of Kazakhstan, with the confirmed presence of *P. betae*. A commercial BNYVV detection system and custom loop-mediated amplification (LAMP) primers were used to confirm the presence of BNYVV. Complete sequences of RNA-3 and RNA-4 cDNA were obtained from 14 selected samples for comparison with sequences available in public databases, including isolates Kas2 and Kas3 previously identified in Kazakhstan (Koenig et al., 1997). Additionally, BCV2 was detected in beet samples infected with BYNVV, and its partial RNA genome sequence was obtained for the first time in Kazakhstan. The obtained results will potentially contribute to understanding of the BNYVV and BCV2 genetic diversity and fill some knowledge gaps due to very limited data on BCV2.

## 2 Materials and methods

### 2.1 Sample collection and nucleic acid isolation

Sixty three root samples of two sugar beet varieties, Viorika KWS (Germany) and Ardan (France), were collected from two separate fields in sugar beet production regions near Taldykorgan city, Eskeldi district, Zhetysu region (former part of the Almaty region), Kazakhstan. Field 1 (area 15 ha; Viorika KWS is grown) was located near Karabulak settlement (44.92111° N, 78.50353° E). Field 2 (area 10; Ardan is grown) ha was located near Malogorovka village (44.93345° N, 78.61747° E). The distance between two field was 9.1 km. Both fields were reported by the farmers for decrease in yield quality and amount.

Prior to sampling, the fields were tested for the presence of *P. betae* (see details below). In the absence of visually noticeable symptoms, asymptomatic roots were collected. Samples were washed from the soil with water, and root hairs were isolated and stored at −80°C until further use. Fresh rootlets from arbitrarily selected samples were wet mounted in the standard PBS buffer solution with the addition of 10% glycerol and 0.05% methylene blue for the miscroscopic examination for the presence of *P. betae* in root tissue.

Soil samples were collected randomly from the same locations as the root samples and stored at −20°C until further use. “Meta soil” metagenomic DNA extraction kit (Raissol Bio, Russian Federation) was used to extract DNA from soil samples following manufacturer’s recommendations. The quantity and quality of the DNA extracts were checked by electrophoresis on a 1% agarose gel in Tris-acetate buffer and using a Nanodrop spectrophotometer (Thermo Fisher, United States) and diluted with water to the working concentration 20 ng/μL.

For RNA extraction, 250 mg of plant material was frozen in liquid nitrogen and ground in 1.5 mL microtubes using sterilized microtube pestles. A Plant/Fungi Total RNA Purification Kit (Norgen Biotech, Canada) was used for total RNA extraction following the manufacturer’s protocol. The quantity and quality of the obtained RNA were checked by electrophoresis on a 1% agarose gel with Tris-acetate buffer and by using a Nanodrop spectrophotometer (Thermo Fisher Scientific, United States). RNA extracts were diluted with water to the working concentration 50 ng/μL.

Reverse transcription (RT) of obtained RNA extracts was performed for an hour at 45°C using Superscript IV reverse transcriptase (Thermo Fisher, United States), according to manufacturer’s protocol. A combination of oligo-dT and random hexameric primers were used. The obtained cDNA samples were further used for sequencing of BNYVV and BCV2 and LAMP assay for BNYVV.

### 2.2 Detection of *Polymyxa betae* in soil samples

Real-time PCR detection of *P. betae* was conducted using the primers and probes provided by LetGen Biotech (Turkey) (Table 1). The reaction mix was prepared using Luna Universal Probe qPCR Master Mix (NEB, United States) for a volume of 20 μL and contained 0.5 μM each of forward and reverse primers, 0.25 μM probe, and 40 ng of prepared DNA extracts from soil samples (2 ng/μl final template concentration). PCR was carried out using Bio-Rad CFX 96 system and the following program: initial denaturation at 95°C for 1 min; 45 cycles of denaturation at 95°C for 15 s, followed by combined annealing and elongation at 58°C for 30 s with measurements of the signal during this step. Parameters of real-time PCR detection were supplied by the provider: Limit of Detection (LOD) 10 copies/μL (estimated by standard deviation); Cq = 37.2 ± 0.5. The validation default settings, i.e., single threshold for the Cq determination mode or up to 500 RFU, automatic baseline subtraction by the supplied Bio-Rad CFX standard software and specific fluorophore for assay were used.

**Table 1:** PCR primers used in the work.

Assay	Primer ID	Primer positions (with NCBI accession numbers)	Primer sequence 5′ > 3′
Detection of *Polymyxa betae,* real-time PCR	PB-F	HE860064.1, 338–358	ATCATGTCGGCAACCGAAAGT
	PB-R	HE860064.1, 395–416	TCTGAGATCTTGTATGGTTCGG
	PB probe	HE860064.1, 362–388	TCGGATTCTTGGAACGATAATCCGCCA
Amplification of BNYVV RNA-3 for sequencing	RNA3-1F	NC_003516.1, 32–51	TTATTTACCCTCAGTTGGTG
	RNA3-1R	NC_003516.1, 1,724–1,743	CTGGTACATTTCACACCCAG
Amplification of BNYVV RNA-4 for sequencing	RNA4-1F	NC_003517.1, 51–70	CAATTCCAGTTGTTTGTCTG
	RNA4-1R	NC_003517.1, 1,394–1,443	CAGTACATCTCAACCTTAAC
Amplification of BNYVV RNA-5 for sequencing	RNA5-1R	NC_003513.1, 18–37	GTTCTAAGTGACGTAAGTGG
	RNA5-1R	NC_003513.1, 1,266–1,285	CACATTTCACATCCAGTCAG
Amplification of BCV2 dsRNA-1 for sequencing	BCV2_rna1_f2	NC_038846.1, 216–237	CGCAAAGAAGGAGTTGTTAAGC
	BCV2_rna1_r2	NC_038846.1, 1,531–1,550	CCCGATTGAGGTATGGATGC
Amplification of BCV2 dsRNA-2a for sequencing	BCV2_rna2a_f1	NC_038845.1, 151–170	AAGTTTCACGGGACTCAACC
	BCV2_rna2a_r1	NC_038845.1, 1,535–1,553	GCCTGCTGCCCTTTATCTG
Amplification of BCV2 dsRNA-2b for sequencing	BCV2_rna2b_f2	NC_038847.1, 164–183	GACTCAAACGTCCTGCTCTT
	BCV2_rna2b_r2	NC_038847.1, 1,454–1,472	GCCTGCTGCCCTTTTATGC

### 2.3 Real-time PCR detection of BNYVV

For the detection of BNYVV, a Real-Time PCR Detection Kit provided by LetGen Biotech, Turkey (Cat# LSK471-0500) was used. This kit was designed for the direct detection of viral RNA by reverse transcription and qPCR within a single tube and includes two reaction mixes: first, for the detection of pathotype B with an FAM-labeled probe, and second, for the detection of pathotypes A and/or P with probes labeled with FAM and Cy5, respectively. The reaction mix was prepared following manufacturer’s recommendation: qPCR MasterMix, 10 μL/sample; primer and probe mix, 2 μL/sample; ultrapure water, 3 μL/sample; RTase Mix, 1 μL/sample; RNA sample, 4 μL per reaction (total RNA concentration 50 ng/μl); and final volume of 20 μL. Negative (water) and positive (included in the kit) controls were used for each qPCR assay. PCR program included: reverse transcription step at 50°C for 10 min; initial denaturation at 95°C for 2 min; 40 cycles of denaturation at 95°C for 10 s followed by combined annealing and elongation at 60°C, with measurements of the signal for 30 s. PCR was run in Bio-Rad CFX 96 real-time amplification system. A sample was considered positive for BNYVV types A, B, or P if the samples satisfied detection criteria according to the provider: LOD 12 copies/μL; Cq 35.0. The validation default settings, i.e., single threshold for the Cq determination mode or up to 500 RFU, automatic baseline subtraction and specific fluorophore for assay were used. PCR run was considered successful if the negative and positive control samples resulted in the expected outcomes.

### 2.4 BNYVV sequencing analysis

Primers for the amplification of RNA-3, RNA-4, and RNA-5 were developed based on the reference genome sequence of BNYVV (NCBI RefSeq accession GCF_000854885.1, isolate S, Japan) using the Primer3 tool and additionally checked for specificity using the Primer BLAST tool (Ye et al., 2012). Amplification was performed in 25 μL reaction mix containing 12.5 μL LongAmp Taq DNA Polymerase Master Mix (New England Biolabs, United States), 1 μL of each primer (10 mM) and 2 μL cDNA from beet samples; the amplification program consisted of 30 s initial denaturation at 94°C, 30 PCR cycles (20 s detaturation at 94°C; 40 s annealing at 51°C; 1 min 40 s elongation at 65°C), and 15 min of final elongation at 65°C. Amplification was confirmed by agarose gel electrophoresis and quantified using a Qubit fluorometer with a Qubit dsDNA Broad Range Kit (Thermo Fisher Scientific). Amplicon sequencing was performed using the Oxford Nanopore MinION Mk1B system with an FLO-MIN114 R10 flow cell. The sequencing library was prepared following the SQK-RBK114-96 (Rapid Barcoding) protocol in accordance with the manufacturer’s recommendations. The amplicons from individual isolates were combined and multiplexed using one barcode per isolate. Base calling and quality control were performed using Nanopore’s Dorado 7.3.9 software using a fast model. The reads obtained from each sample were mapped against the reference BNYVV genome sequence (GCF_000854885.1) using the BWA MEM tool with the Nanopore read compatibility option (−ont2d) (Li, 2013). Mapping statistics and consensus sequences for each sample were obtained using SAMTools v.1.19 (Li et al., 2009). After the sequences from each isolate were obtained by calculating consensus sequences from the reads mapped against the reference genome, they were aligned between each other and the corresponding reference genomic sequences using MAFFT (Katoh et al., 2002) and trimmed to exclude low-quality terminal sequences.

A National Center for Biotechnology Information (NCBI) nucleotide database search was used to obtain a set of previously published sequences of BNYVV RNA-3 and RNA-4. All available accessions covering the obtained RNA-3 or RNA-4 sequences were selected. The MAFFT tool was used to perform multiple sequence alignments. The alignment was filtered and trimmed to include only sequences corresponding to the target region. MEGA v.11 software (Tamura et al., 2021) was used to calculate the maximum likelihood tree with 500 bootstrap replications using Tamura-Nei nucleotide substitution model with uniform rates among sites. The calculated trees were rooted at the midpoint.

RDP5 (Martin et al., 2021) software was used for recombination analysis of BNYVV RNA-3 and RNA-4. The same set of sequences was used for recombination analysis as for phylogenetic tree construction.

### 2.5 BNYVV loop mediated isothermal amplification

LAMP primers for BNYVV detection were designed using the NEB LAMP Primer Design Tool (New England Biolabs, United States) (Table 2). All available sequences for complete RNA2 of BNYVV were retrieved from NCBI (accessed on 10 December 2023). MUSCLE method was applied to multiple sequence alignment (Edgar, 2004) and identification of conserved region which was used in NEB LAMP Primer Design Tool.

**Table 2:** Primers used for LAMP detection of BNYVV.

BNYV-F3-2-001	TATGACCGATCGATGGGC
BNYV-B3-2-002	TAACACAAGTGCACCGTA
BNYV-FIP-2-003	AGATAGATTCGCAGCCTTGGACGTCGTGAGTGTTATTAAACAATC
BNYV-BIP-2-004	GCTCGGGTTGGACTGACAATAGTGTCTGTGGAAAACGG

The coat protein gene sequence from the reference BNYVV genome (Japanese isolate S (Saito et al., 1996), RefSeq ID GCF_000854885.1; segment RNA-2 NC_003515.1) was used as the target for developing chimeric positive control. The target region included nucleotide positions 173–473 in the reference RNA-2 sequence or position 29–329 of CP ORF and was cloned into pMG-Amp plasmid. The plasmid pMG-Amp with the target sequence was ordered from Macrogen (Seoul, Korea). LAMP was performed using WarmStart Colorimetric LAMP 2X Master Mix with UDG (New England Biolabs, United States). Reaction mixes included 12.5 μL 2x LAMP master mix, 2.5 μL LAMP primer mix (FIP 16 μM, BIP 16 μM, F3 2 μM, B3 2 μM), 1 μL sample cDNA, and ddH2O to the final volume 25 μL. Master mix contained indicator phenol red; the change of the reaction mix color from, red to yellow was expected for positive reactions. The first reaction was performed using chimeric control DNA diluted in series from 1 ng to 1 fg to optimize and evaluate sensitivity thresholds. The reaction tubes were incubated at 65°C for 45 min to record the color of the mixtures and take 5 μL for electrophoresis in 1% agarose gel with standard TAE buffer every 15 min. After preliminary optimization, LAMP was tested on all cDNA samples from beet specimens that tested positive and negative for BNYVV by real-time PCR. The results were recorded after 45 min of incubation at 65°C.

To evaluate non-specificity of developed primers for LAMP assay, virus-free plants of 14 lines of sugar beet were tested (Table 3). The plant seeds were planted in sterile soil under control conditions in climatic chamber (Binder KBWF 240, Tuttlingen, Germany). To ensure the absence of BNYVV, the 3-week-old plants were tested by real-time PCR. Further, cDNA of each sample was used for LAMP assay. Additionally, cDNAs of genomic segments of BCV2 identified and amplified by RT-PCR in the present work (see section below) were used for non-specificity test. The conditions for amplification were described above.

**Table 3:** The sugar beet lines tested for non-specific amplification using the LAMP assay.

#	Hybrid	Origin
1	Klima	France
2	22b5006	France
3	FD Bunker	France
4	Eider	France
5	22b5004	France
6	Puls	France
7	Pamyati abugalieva	Kazakhstan
8	Aksu	Kazakhstan
9	Abulhair	Kazakhstan
10	Bolashak	Kazakhstan
11	Taraz	Kazakhstan
12	Concertina	Germany
13	Viorika	Germany
14	Alando	Danmark

### 2.6 BCV2 detection and analysis

Primers for the amplification of dsRNA-1, dsRNA-2a, and dsRNA-2b were developed based on the reference genome sequence of BCV2 (NCBI RegSeq accession GCF_002868515.1, isolate S, Hungary) using Primer3 and Primer BLAST tools (Table 1). Amplification was performed in 25 μL reaction mix containing 12.5 μL LongAmp Taq DNA Polymerase Master Mix (New England Biolabs, United States), 1 μL of each primer (10 mM) and 2 μL cDNA from beet samples; the amplification program consisted of 30 s initial denaturation at 94°C, 30 PCR cycles (20 s denaturation at 94°C; 30 s annealing at 58.5°C; 75 s elongation at 65°C), and 10 min of final elongation at 65°C. Sequencing and reading were performed as described above for BNYVV. The obtained sequences were compared with those of isolates available in the NCBI GenBank database using MAFFT multiple sequence alignment.

## 3 Results

### 3.1 BNYVV detection and sequencing

**Figure 1:**
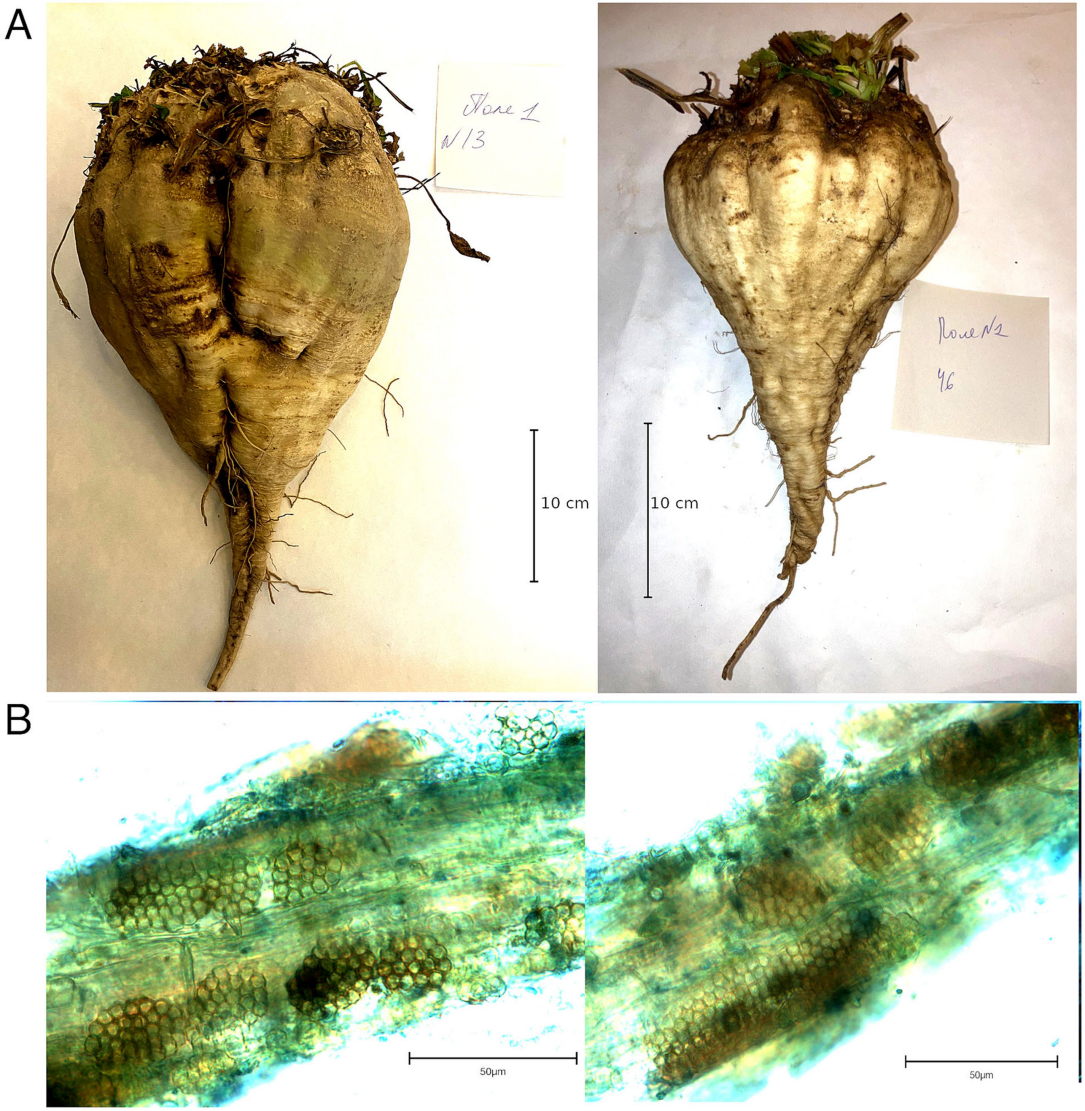
(A) Beet roots collected from field 1 (left) and 2 (right); (B) *Polymyxa betae s*porosori within beet roots.

The two sampling locations were tested for the presence of *P. betae* prior to sample collection. As a result, 47 of 55 soil samples collected from Field 1 and 47 of 53 samples from Field 2 were tested positive for the vector (85.5 and 88.7%, respectively), indicating wide infestation coverage in these areas. Visual examination of living sugar beet plants revealed no characteristic symptoms, yellowing, necrotic lesions of the above-ground parts, or anomalous root growth (Figure 1A); the only distinct trait observed was smaller beet root size in some samples. Asymptomatic samples were collected for the detection of BNYVV. Microscopic examination confirmed the presence of the BNYVV vector, *Polymyxa betae,* in the root tissue of the collected samples (Figure 1B).

A total of 123 samples were tested for the presence of BNYVV using real-time PCR. Of the 63 samples from field one (cultivar Viorika KWS), 49 tested positive for A type, and 58 of the 60 samples from field two (cultivar Ardan) tested positive for A type; no samples produced reliable signals for BNYVV types B and P.

**Figure 2:**
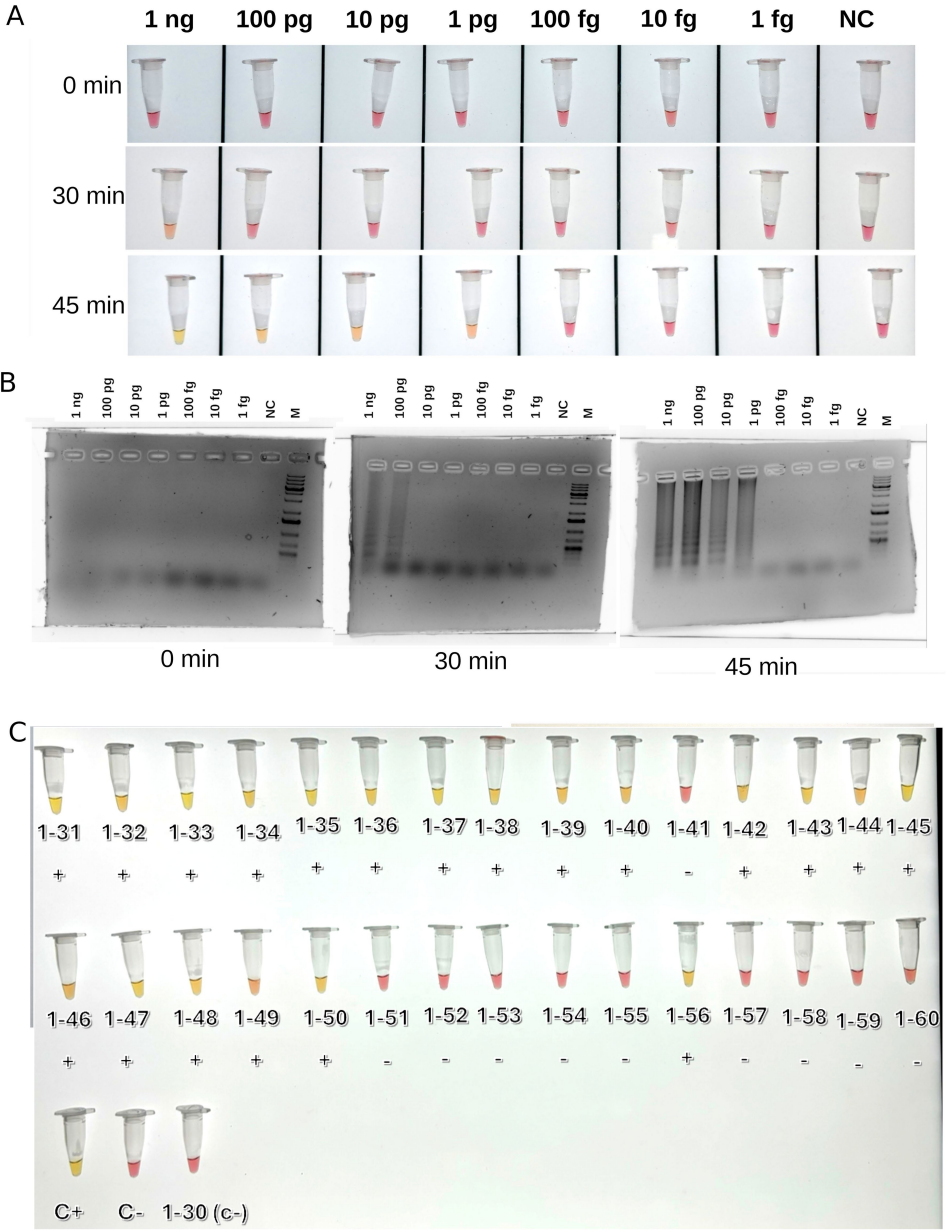
Results of LAMP detection of BNYVV. (A) Test reactions with series of dilutions o the control template (plasmid); (B) Agarose gel electrophoretic images of test reactions; the presence of the series of smeared bands indicates positive LAMP reaction; (C) LAMP reactions on BNYVV on beet samples, after 45 min incubation. C+, positive control (chimeric plasmide carrying target sequence); C−, negative control (water); M, Thermo Fisher 1 Kb Plus DNA Ladder. Signs below samples indicated outcomes of real-time PCR based detection (negative or positive).

A set of primers targeting the BNYVV coat protein gene sequence was developed for LAMP. Preliminary testing of a series of dilutions of the control template (plasmid with the target sequence) showed that detection was possible for target amounts as small as 1 pg. per reaction (Figures 2A,B). Testing of all samples from the present research, as demonstrated in Figure 2C and Supplementary Figure S1, provided results that were fully consistent with those obtained from real-time PCR analysis. This consistency reinforces the reliability of the developed primers for LAMP detection as a fast and efficient alternative to PCR-based detection techniques. The non-specificity analysis revealed no cross-reaction with other sequences. This analysis involved testing the primers against non-target DNA such as DNA from noninfected sugar beet plants (14 hybrids) and cDNAs of BCV2 isolates detected in present work to ensure that the amplification process did not produce false-positive results (Supplementary Figure S1). A total of 14 positive BNYVV samples were arbitrarily selected for sequencing analysis, including seven isolates from each of studied fields (Table 4). PCR for RNA-3 and RNA-4 produced fragments of the expected size with nearly complete sequences, whereas the primers for RNA-5 failed to produce any result. As a result, partial sequences of RNA-3 and RNA-4 with lengths of 1,695 and 1,361 bp, respectively (comparing to complete sequences lengths of 1774 and 1,465 bp, respectively, according to the reference BNYVV genome GCF_000854885.1), were obtained and compared with isolates from different regions of the world. The obtained sequences of RNA-3 and RNA-4 contained complete ORFs encoding the proteins p25 and p31, respectively.

**Table 4:** Summary of nanopore sequencing results of RNA-3 and RNA-4 of BNYVV, dsRNA-1, dsRNA-2a, dsRNA-2b of BCV2.

Virus	Isolate	Total bases, Mb	Passed bases, %	Total reads, thousands	Passed reads,%	Reads aligned against reference, thousands	Average coverage depth (RNA-3, 4, or dsRNA-1, 2a, 2b)
BNYVV	Kz1-3	11.91	93.8	17.42	91.4	15.70	2,495, 4,288
	Kz1-32	11.81	95.5	16.19	93.4	14.95	2,947, 3,916
	Kz2-41	18.88	94.2	29.85	91.6	26.67	4,652, 6,087
	Kz2-51	19.87	94.9	28.75	93	26.01	5,462, 5,734
	Kz1-49	4.91	93.5	8.39	88.3	6.37	1,401, 1,180
	Kz2-01	11.05	95.1	16.33	92.5	14.78	2,597, 3,800
	Kz1-10	11.53	94.9	17.19	91.4	15.47	2,611, 4,082
	Kz1-16	17.09	94.7	24.26	92.8	22.14	4,184, 5,664
	Kz2-10	3.51	90.6	7.35	83.7	4.44	1,044, 577
	Kz2-05	15.92	95.6	22.99	93.4	20.55	3,339, 5,899
	Kz1-28	13.6	95.4	20.09	93.8	18.53	3,471, 4,349
	Kz2-36	11.99	93.9	18.47	91.2	15.52	2,950, 3,660
	Kz2-58	24.5	94.9	34.47	93.3	31.35	7,344, 6,457
	Kz1-8	27.62	94	40.21	92.1	36.35	7,018, 8,654
BCV2	Kz2-41C	16.88	94	24.88	92.5	22.38	4,652, 4,035, 968
	Kz1-49C	16.15	93.8	23.84	92	21.36	720, 4,193, 4,474
	Kz2-01C	43.04	93.1	69.24	91.7	62.09	6,026, 12,814, 5,544
	Kz2-10C	13.34	94	19.02	92.4	17.42	3,022, 2,596, 2,165
	Kz2-36C	33.45	92.1	52.7	91.5	43.66	7,786, 1,495, 9,303
	Kz2-58C	14.68	92.8	25.99	89.6	16.26	1,900, 893, 4,833

**Figure 3:**
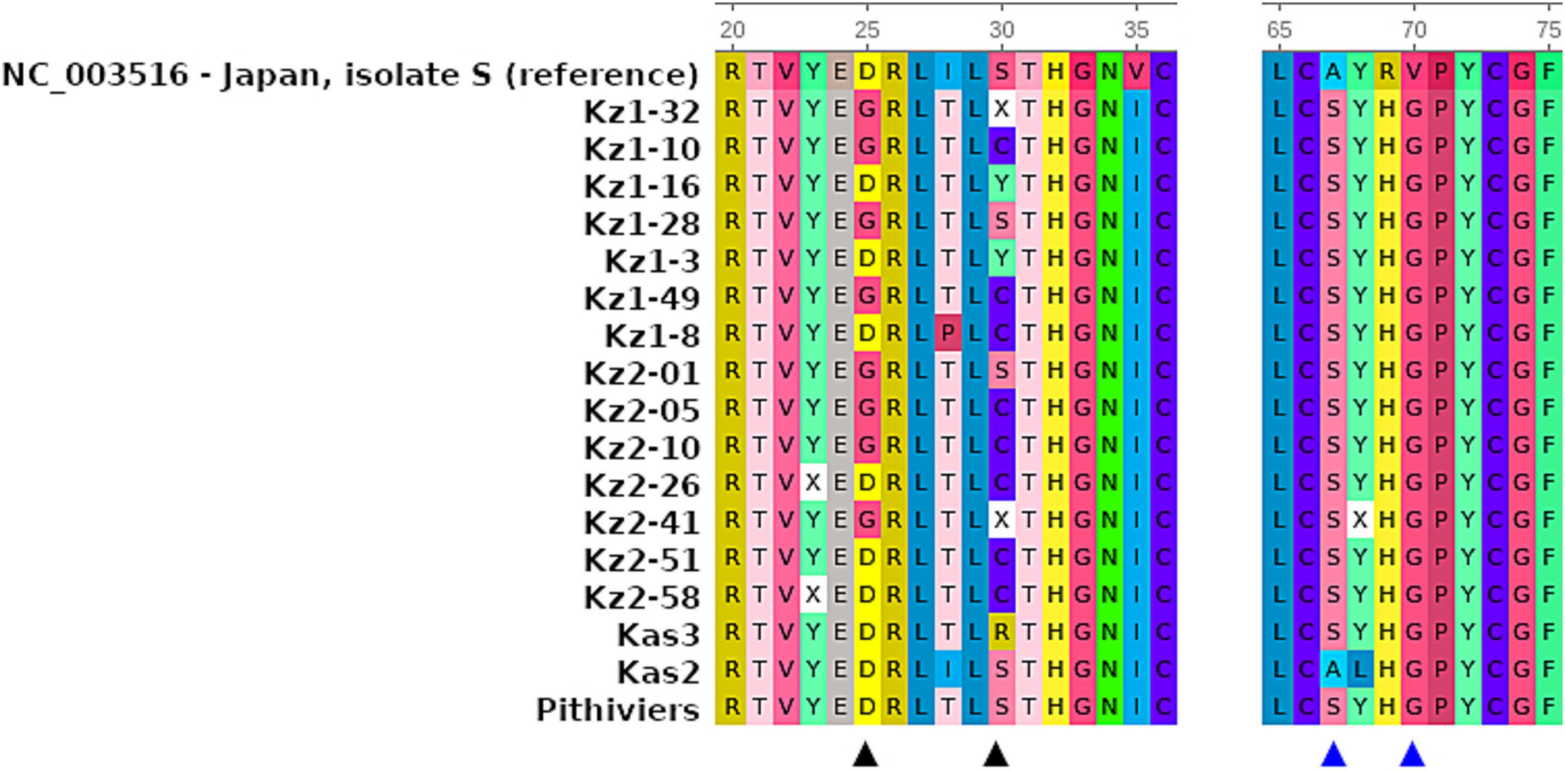
Fragments of multiple sequence alignments of highly variable positions and tetrade SYHG within p25 protein sequences of 14 BNYVV isolates from Kazakhstan, in comparison to the reference sequence and previously studied isolates Pithiviers, Kas2, and Kas3. The ruler indicates amino acid positions relatively to the complete protein sequence. Amino acid colors according the built-in UGENE protein sequence coloring scheme. Black triangles indicate variable positions in studied sequences; blue triangles indicate amino acid tetrade 67-70.

RNA-3 sequences had missing parts of 39 and 41 bp at the 5′ and 3′ termini, correspondingly. All the RNA-3 sequences obtained were highly similar (Supplementary Figure S2). Variable positions were observed between nt 519 and 543 (hereafter, base positions are indicated relative to the reference sequence) within the ORF of the p25 protein. At position 519, eight of the 14 isolates had an A > G substitution, which was not observed in either the reference or any other sequences used for comparison. This resulted in an Asp25Gly substitution in the p25 amino acid sequence (Figure 2). Ten of the fourteen isolates had a substitution A > T at position 533, which is unique to our isolates. All 14 isolates harbored a C > T substitution at position 535 which was rare across sequences used for comparison. Amino acid position 30 resulting from nucleotide variants 533–535 displayed variable cysteine or tyrosine substituting the more common serine (Figure 3; Supplementary Figure S2). With the exception of the mentioned variations, the obtained sequences were nearly identical to each other and isolate Kas3, and were highly similar to the isolate Pithiviers (France), as can be seen from the multiple sequence alignment (Supplementary Figure S2A). The amino acid tetrade at protein positions 67–70 was SYHG, as well as in the Kas3 and Pithivier isolates (Figure 3). No systematic differences were found between two sampled fields.

**Figure 4:**
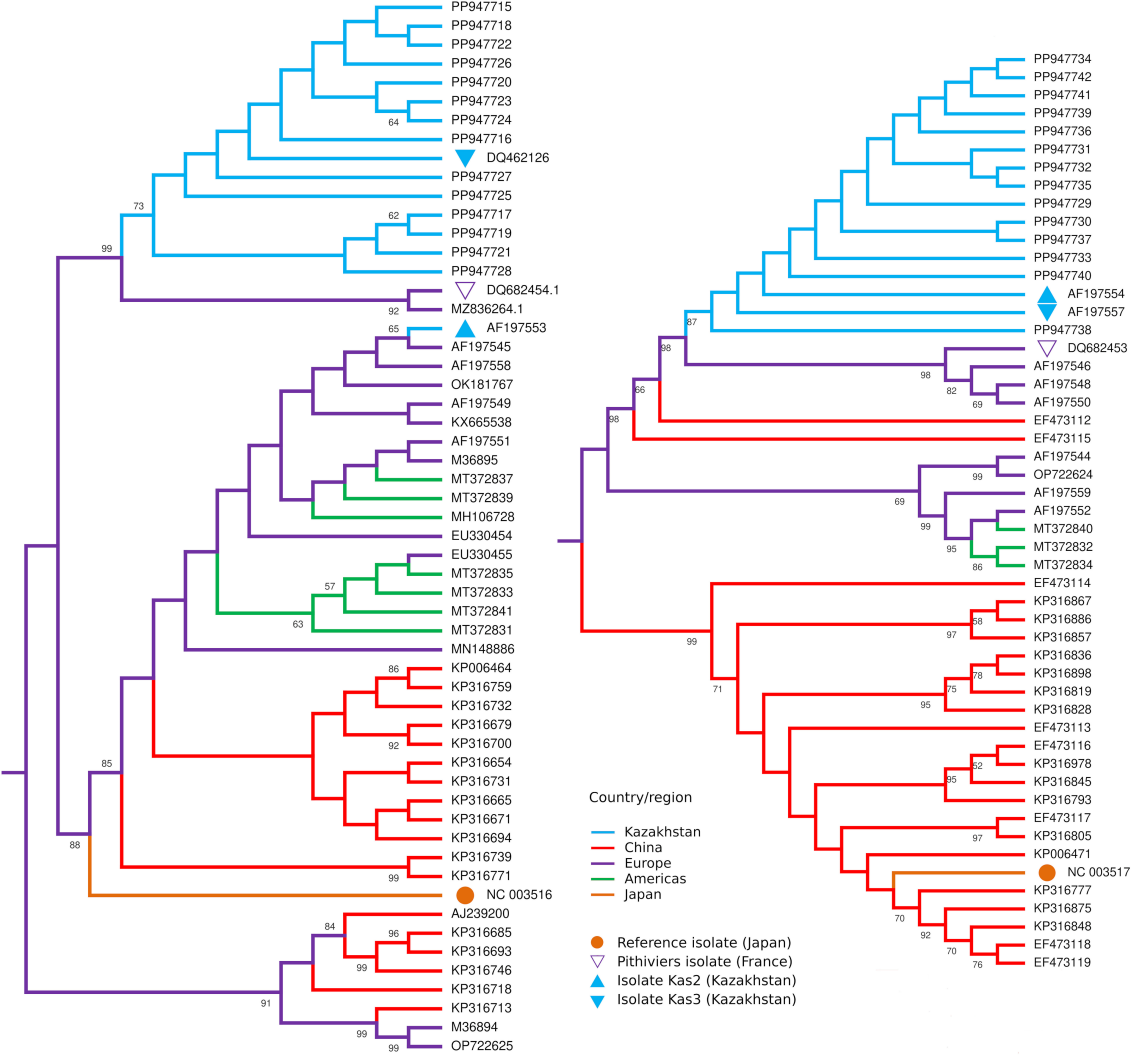
Midpoint rooted maximum likelihood trees of the partial RNA3 (left) and RNA4 (right) sequences of BNYVV isolates from Kazakhstan (blue) in comparison with isolates from other countries. Numbers at the nodes display bootstrap values above 50%.

RNA-4 sequences had missing parts of 43 and 62 bp at the 5′ and 3′ termini, correspondingly. All 14 sequences were nearly identical to the corresponding sequences of isolates Kas2, Kas3, and Pithiviers, except for occasional ambiguous sites (Supplementary Figure S3). The maximum likelihood tree for RNA-4 (Figure 4) included isolates Kas2 and Kas3 clustered along with 14 new sequences, whereas in the tree for RNA-3 isolate Kas2 was excluded from this group; proximity to the Pithiviers isolate was retained in both trees.

The search for the possible recombination event in RNA-3 and RNA-4 using RDP5 software has shown no results from any of the available algorithms.

### 3.2 BCV2 sequencing

The 14 beet root RNA samples used for the detection and sequencing of BNYVV were screened for BCV2 using custom primers (Table 1). Six samples were positive for the virus and produced large fragments of all three dsRNA segments suitable for sequencing, including one isolate from field 1 and five isolates from field 2. The obtained consensus sequences of dsRNA1, dsRNAa, and dsRNAb were trimmed to exclude low-quality terminal parts with final lengths of 1,318, 1,388, and 1,298, respectively (corresponding reference sequences lengths 1,575, 1,598, and 1,522). The total genome coverage of BCV2 was 85.28%. All three segments had only partial coding sequences of corresponding ORFs trimmed at 5′ termini. A search of the BCV2 RNA sequences in the NCBI GenBank database identified only two complete isolates, including the reference genome (GCF_002868515.1). These isolates were obtained from Hungary and Germany. Therefore, comparisons of the obtained sequences are limited.

Sequences of dsRNA1 had missing parts of 222 and 35 bp at the 5′ and 3′ termini, correspondingly, relatively to the reference. All identified sequences were nearly identical among the isolates, except for occasional ambiguities. The sequences were highly similar to those of the Hungarian isolate, with the exception of the ambiguous position 1,463 (relative to the reference). The German isolate differed by substitutions at positions 232, 433, and 944 (Supplementary Figure S4A).

Sequences of dsRNA2a had missing parts of 147 and 53 bp at the 5′ and 3′ termini, correspondingly. All identified sequences were identical between the isolates and the Hungarian isolates. The German isolate differed in substitutions at five positions (478, 538, 547, 1,030, and 1,113) (Supplementary Figure S4B).

Sequences of dsRNA2b had missing parts of 167 and 57 bp at the 5′ and 3′ termini, correspondingly. All sequences contained more ambiguous positions than dsRNA1 and dsRNA2a; in particular, the most ambiguous positions were found in the sample Kz1-49C. Three of the six samples had adenine at position 173 (relative to the reference) in comparison to guanine at this position in Hungarian and German isolates. This position was the only confirmed difference between the BCV2 samples and the reference strain (Supplementary Figure S4C).

## 4 Discussion

To date, the only available data on BNYVV sequences from Kazakhstan were published in 2000 (Koenig and Lennefors, 2000). In that study, two distinct isolates, Kas2 and Kas3, were identified. Isolate Kas2 possessed RNA-5 similar to the Pithiviers isolate; however, the RNA-3 sequence was more similar to that of the A type (Ward et al., 2007). Isolate Kas3 had an RNA-3 sequence highly similar to that of the Pithiviers isolate, but lacked RNA-5 (Koenig and Lennefors, 2000). The authors of previous studies noted a peculiar similarity between isolates from distant locations such as France and Southeast Kazakhstan with little known connections and hypothesized that the virus of the P-type could be introduced to both countries independently from an unknown third country (Koenig and Lennefors, 2000). Here, we attempted to update existing knowledge regarding the presence of BNYVV in Kazakhstan. Our study relied on the real-time PCR identification of the virus, followed by RNA-3 and RNA-4 sequencing of selected samples. The custom PCR detection kit by the LetGen Biotech company (Turkey) uses the coat protein-coding sequence of RNA-2 for differential detection of A and B types, and the p26 protein-coding sequence of RNA-5 for detection of P-type viruses; however, the exact targets for primers and probes have not been disclosed by the provider. Two fields from which the infected plant material was sampled were investigated after farmers reported unexplained yield losses (private communication). Both real-time PCR assays on soil samples and microscopic examination of beet roots revealed wide-scale infestation by *P. betae* at the sampling locations. Despite the absence of visual rhizomania symptoms, real-time PCR tests were positive for most beet samples. Real-time PCR indicated the presence of BNYVV with the A type coat protein.

In addition to real-time PCR, we developed a new set of primers for the LAMP-based detection of BNYVV. Until now, most detection systems for BNYVV were based on immunoassay or PCR methods (Sukhacheva et al., 1996; Morris et al., 2001). Immunoassays offer reliable diagnostic results but are limited by the affinity of antigen–antibody interactions and potential cross-reactivity. In contrast, nucleic acid amplification methods, such as PCR, provide highly sensitive and specific detection. However, PCR requires laboratory conditions and specialized equipment, making it unsuitable for field applications. In addition to traditional nucleic acid detection methods, isothermal amplification techniques including LAMP have emerged as valuable alternatives. The LAMP offer the advantage of amplifying nucleic acids at a constant temperature, providing a more versatile and rapid approach for diagnostics, particularly in field-based and resource-limited settings and does not require complicated equipment for running and registering results (Notomi et al., 2015). The only LAMP assay for the detection of BNYVV comprising FIP, BIP, F3, and B3 primers was previously developed by Almasi and Almasi (2017). These primers were developed for the coat protein gene on the RNA2 segment of BNYVV isolate Pithivier (P-strain) and used for testing sugar beet samples collected in 10 fields located in Azerbaijan and Iran (Almasi and Almasi, 2017). In the present study, primers were designed for the conserved region of coat protein using all available sequences of the complete RNA2 segment of BNYVV, including A, B, and P- type strains, retrieved from the NCBI database. These primers are expected to be highly effective in detecting the aforementioned BNYVV strains and were successfully tested on the A-type strain identified in the present work. The test reaction in the present study showed that LAMP allowed the detection of a small amount of template DNA (1 pg. per reaction within 30–45 min). The results of the test on cDNA from the beet samples conformed to the real-time PCR output. Furthermore, no cross-reactivity of the developed primers with cDNA from various sugar beet hybrids or BCV2 was observed. Thus, the developed LAMP primers are suitable for the rapid testing of beets, which is important for the timely detection, control, and management of the virus.

Following the real-time PCR based identification, we attempted the sequencing analysis of BNYVV genome segments RNA-3 and RNA-4. These segments, especially RNA-4 encoding p25 protein, not detected in our study provide high level of variability allowing to detect more detailed phylogenetic structure than RNA-2 with CP gene (Tamada et al., 2016). Moreover, these RNA species contribute to the transmission and symptom development (Peltier et al., 2008) and thus their variability is important for disease severity and its propagation. RNA-5, which also significantly affect infection process, was not detected either by the real-time PCR detection kit or custom primers, similarly to isolate Kas3 (Koenig and Lennefors, 2000). Analysis of RNA-3 sequences showed high similarity between the obtained samples and isolates Pithiviers and Kas3 and a notable distinction from isolate Kas2. Considering the absence of RNA-5, Kas3 was the most closely related isolate. An intriguing signature of the local BNYVV samples was the presence of variations at positions 25 and 30 of the p25 protein. Substitution Asp25Gly occurred within the two sampled populations, despite the relative conservation of this position, based on the examined sequences (Supplementary Figure S2). A rare substitution of serine with cysteine or tyrosine was observed at position 30. Such variations imply the presence of multiple haplotypes of the virus in the same fields in Kazakhstan. RNA-3 is known to be responsible for the expression rhizomania symptoms (Tamada et al., 1999), thus the discovered variants may be related to the lack of the disease symptoms in tested sugar beet plants. Such variation may indicate the persistence of low pathogenic BNYVV strain(s) in the sugar beet growing area of Kazakhstan, however, further conclusions require more extensive studies. Although the samples were collected from the separate fields with the distance between them 9.1 km, these variations were present in both populations. The similarity of isolates from two sampling locations may be explained either by cross-contamination between fields (however, no records of possible interconnections available) or by free persistence of the virus on the wider area covering both sites.

Interestingly, cysteine at position 30 has previously been reported in Iranian BNYVV isolates, similar to type P but lacking RNA-5 (Mehrvar et al., 2009). The authors attributed these isolates to Northern Iran, which is closer to Central Asia. The reported p25 protein sequence features of these proteins were similar to those of isolates from Kazakhstan and Pithivier. Although we have not directly compared our data with the Iranian data due to their incompleteness, the similar variations in RNA-3 segment and the absence of RNA-5 may indicate the existence of a specific BNYVV strain in Central Asia; however, the possibility of similar variants resulted from convergent evolution should also not be neglected. Thus, more data on genetic diversity of BNYVV in this region are required to corroborate either of the hypotheses. To date, most data on the BNYVV sequence variability have covered Europe (Koenig et al., 2008; Koenig and Lennefors, 2000; Ward et al., 2007), China (Zhuo et al., 2015), and the United States (Weiland et al., 2020). Data on two isolates from Kazakhstan, Kas2 and Kas3, published in 2000 and used in numerous subsequent comparative studies, are limited, as we show here based only on two populations. RNA-3 of the virus in Kazakhstan shows some degree of variability. Further studies employing RNA-1 and RNA-2 sequences should be conducted to elucidate the evolution of BNYVV, as segment reassortment is an essential part of this process (Chiba et al., 2011).

For the first time, BCV2 was detected in sugar beet fields in Kazakhstan. Previously, we detected this virus in sugar beet samples using exploratory RNA sequencing (unpublished data). Cryptic or persistent viruses are a family of plant viruses with double-stranded RNA genomes that display absent or weak symptoms and are transmitted only through plant reproduction with seeds or pollen (Boccardo et al., 1987). To date, little is known about beet cryptic viruses, BCV1, BCV2, and BCV3, and their negligible impact on beet culture. However, their potential synergistic effect on other viral infections cannot be ruled out (Xie et al., 1994). Previously, BCV2 was reported to be co-infected with a beet soil-borne virus (*Pomovirus* genus of *Virgaviridae* family) in Germany (Gaafar et al., 2019). As we have found BCV2 in plants infected with BNYVV, this is the first report of BCV2-BNYVV coinfection generally and, specifically, in Kazakhstan. Since sequences for only two BCV2 isolates are available in the GenBank database, our results are an important contribution to the data on BCV2 variability. Interestingly, compared to the reference Hungarian isolate, our isolates differed in only one position in the dsRNA-2b segment, whereas the German isolate was more diverse. Considering the transmission of the virus and the use of foreign sugar beet cultivars in Kazakhstan, we assume that the virus was imported with sugar beet seeds. Although no immediate impact of cryptic viruses on sugar beets is known, their distribution should be monitored along with that of other beet viruses. The possible interaction between BNYVV and BCV2 in infected beet plants should be the subject of further studies, as the present data are insufficient for drawing conclusions on whether the lack of rhizomania symptoms could be attributed to any synergistic effect between BNYVV and BCV2 or not.

The study utilized a conventional PCR amplification of cDNA sequences of BNYVV and BCV2 RNAs following by nanopore sequencing of the obtained amplicons. This technology gets increasing attention as a rapid, efficient, and scalable tool for sequencing of viral genomes (Kilianski et al., 2015; Munro et al., 2023). Previously, we have utilized such approach to obtain genomic sequences of the isolates of the raspberry bushy dwarf virus from Kazakhstan (Kolchenko et al., 2023). As the practice shows, nanopore sequencing becomes more efficient alternative to the traditional Sanger sequencing in the studies of viruses. Although nanopore sequencing is known to have lower accuracy comparing to other sequencing methods, this limitation can be overcome by deep read coverage (e.g., thousands of reads per position in this study, see Table 3).

Our study was the first attempt to identify BNYVV sequence variability in Kazakhstan since the work by Koenig and Lennefors, 2000. Surprisingly, the plant material displaying lack of rhizomania symptoms was in mass tested positive for BNYVV. Based on our studies two possible reasons may be speculated: on the one hand, the low pathogenicity could be associated with base variations in RNA-3 leading to amino acid changes in p25 protein; on the other hand, the lack of symptoms may be caused by the interactions with BCV2 and/or other beet viruses. Anyways, the further studies on beet viruses present in Kazakhstan are required, including wide scale genomic diversity analysis of BNYVV in combination with BCV2 co-infection tests. Nevertheless, the obtained results are important for the knowledge on the diversity of BNYVV and, particularly, poorly studied BCV2.

## Data Availability

The datasets presented in this study can be found in online repositories. The names of the repository/repositories and accession number(s) can be found at: https://www.ncbi.nlm.nih.gov/genbank/, PP947715-PP947742; PP987715-PP987732; https://www.ncbi.nlm.nih.gov/, PRJNA1157397; https://www.ncbi.nlm.nih.gov/, SAMN43512484-SAMN43512503.
