# Supplementary figures and images for: Molecular detection and sequencing of beet necrotic yellow vein virus and beet cryptic virus 2 in sugar beet from Kazakhstan
Source: Front Microbiol. 2024 Nov 12;15:1461988. doi: 10.3389/fmicb.2024.1461988 (PMC11588710; doi:10.3389/fmicb.2024.1461988)

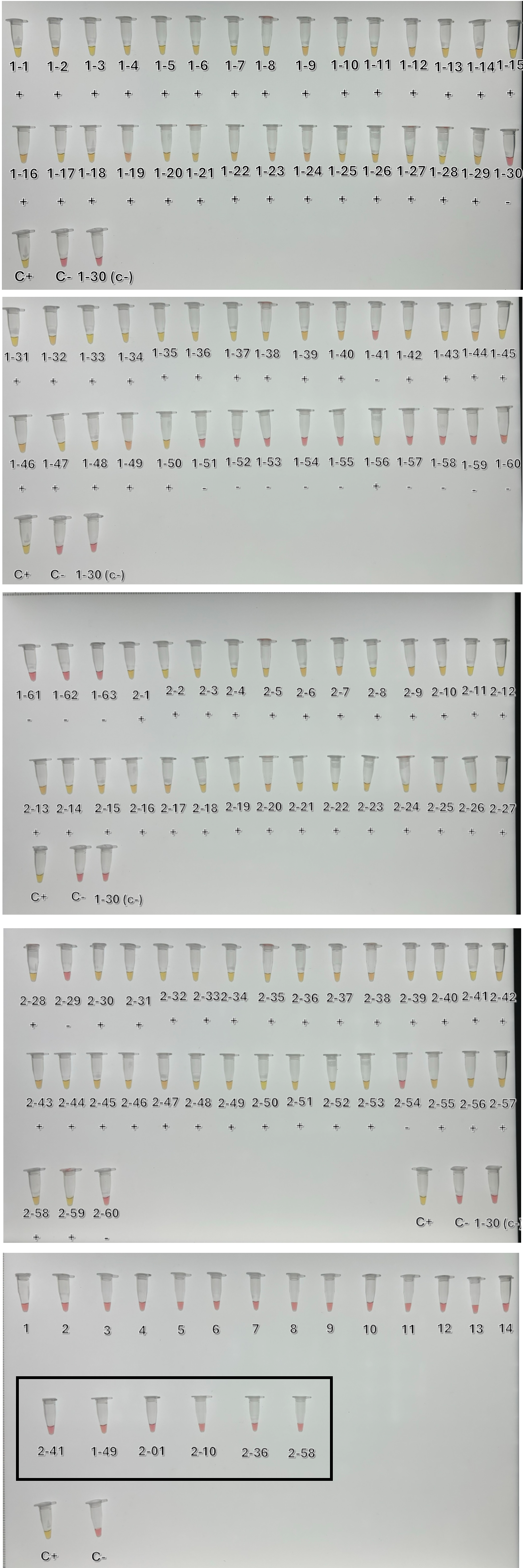

Supplement: Supplementary file 1 [file Image_1.PNG]

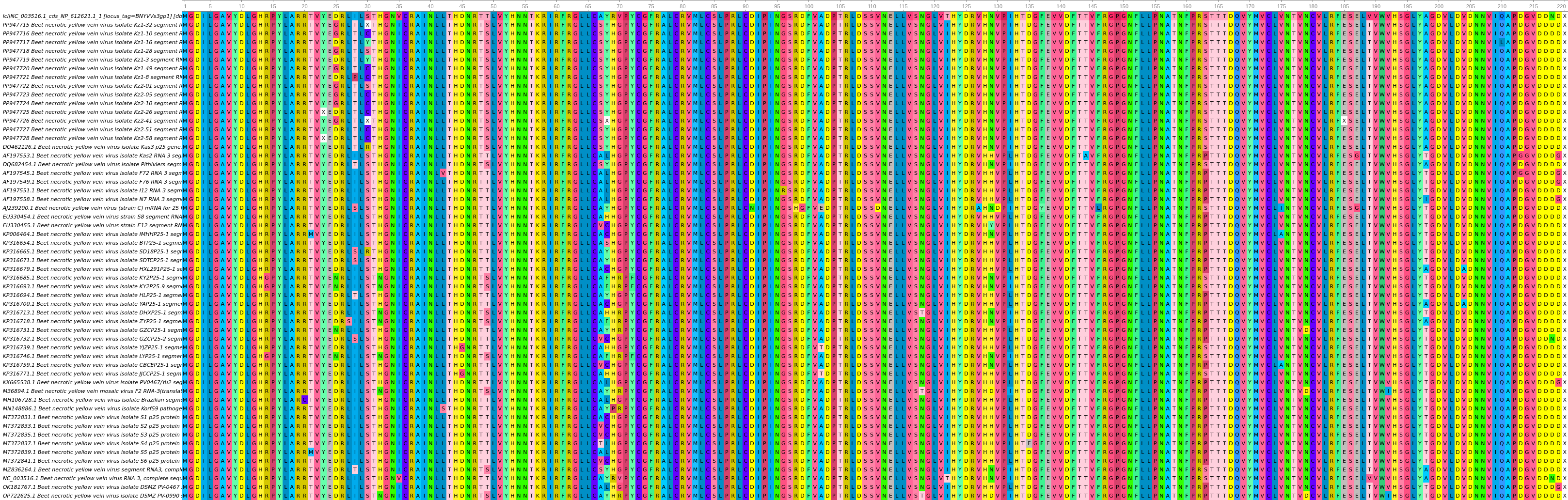

Supplement: Supplementary file 3 [file Image_3.PNG]

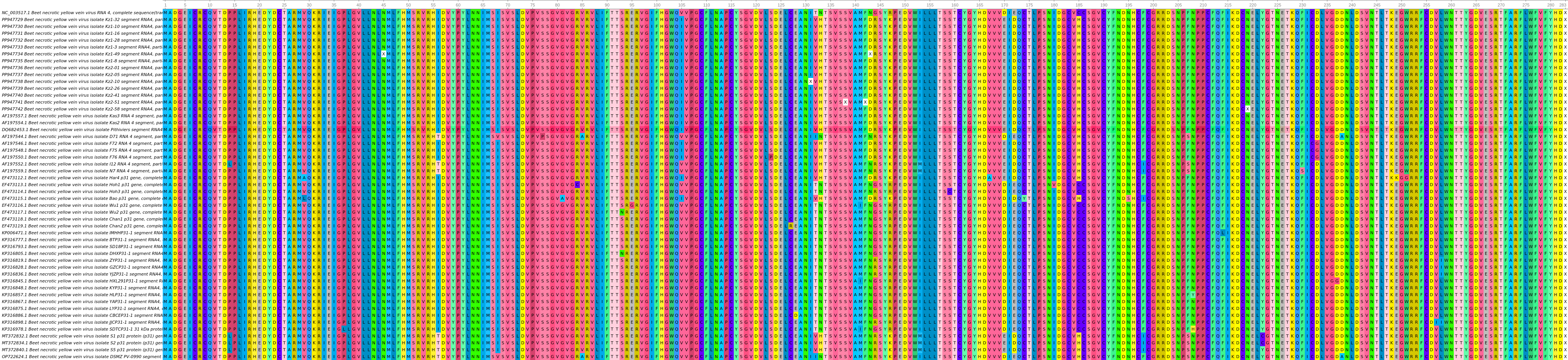

Supplement: Supplementary file 5 [file Image_5.PNG]
