# Supplementary material for: Molecular detection and sequencing of beet necrotic yellow vein virus and beet cryptic virus 2 in sugar beet from Kazakhstan
Source: Front Microbiol. 2024 Nov 12;15:1461988. doi: 10.3389/fmicb.2024.1461988 (PMC11588710; doi:10.3389/fmicb.2024.1461988)
Supplement: Supplementary file 9 [file Table_1.DOCX]

**Supplementary figure 1.** Results of LAMP detection of BNYVV. All samples numbered by field. Samples 1-14 according Table 3. Box indicated amplified cDNA from six BCV2 isolates. Signs below samples indicated outcomes of real-time PCR based detection (negative or positive). C+ - positive control (chimeric plasmide carrying target sequence), C- - negative control (water). Sample 1-31 tested negative was used as additional negative control within independent runs.

**Supplementary figure 2.** Multiple sequence alignment of partial sequences of RNA-3 segment of BNYVV (A) and the translated amino acid sequences of p25 protein (B) in comparison with foreign isolates. The trimmed sequences shown along with the complete reference RNA-3 sequence.

**Supplementary figure 3**. Multiple sequence alignment of partial sequences of RNA-4 segment of BNYVV (A) and the translated amino acid sequences of p31 protein (B) in comparison with foreign isolates. The trimmed sequences shown along with the complete reference RNA-4 sequence.

**Supplementary figure 4.** Multiple sequence alignment of partial sequences of segments dsRNA1 (A), dsRNA2 (B), and dsRNA3 (C) of BCV2 and in comparison with two isolates from Hungary and Germany
